# Diagnostic point-of-care ultrasound (POCUS) for gastrointestinal pathology: state of the art from basics to advanced

**DOI:** 10.1186/s13017-018-0209-y

**Published:** 2018-10-15

**Authors:** Fikri M Abu-Zidan, Arif Alper Cevik

**Affiliations:** 1 Department of Surgery, College of Medicine and Health Sciences, UAE University, Al-Ain, 17666 United Arab Emirates; 2 Department of Internal Medicine, College of Medicine and Health Sciences, UAE University, Al-Ain, 17666 United Arab Emirates

**Keywords:** Ultrasound, Point-of-care, Basic physics, Bowel obstruction, Bowel perforation, Free fluid, Appendicitis, Diverticulitis, Tumour, Intestine, Inflammation, Tuberculosis

## Abstract

The use of point-of-care ultrasound (POCUS) by non-radiologists has dramatically increased. POCUS is completely different from the routine radiological studies. POCUS is a Physiological, On spot, extension of the Clinical examination, that is Unique, and Safe. This review aims to lay the basic principles of using POCUS in diagnosing intestinal pathologies so as to encourage acute care physicians to learn and master this important tool. It will be a useful primer for clinicians who want to introduce POCUS into their clinical practice. It will cover the basic physics, technical aspects, and simple applications including detection of free fluid, free intraperitoneal air, and bowel obstruction followed by specific POCUS findings of the most common intestinal pathologies encountered by acute care physicians including acute appendicitis, epiploic appendagitis, acute diverticulitis, pseudomembranous colitis, intestinal tuberculosis, Crohn’s disease, and colonic tumours. Deep understanding of the basic physics of ultrasound and its artefacts is the first step in mastering POCUS. This helps reaching an accurate POCUS diagnosis and avoiding its pitfalls. With increased skills, detailed and accurate POCUS findings of specific intestinal pathologies can be achieved and properly correlated with the clinical picture. We have personally experienced and enjoyed this approach to a stage that an ultrasound machine is always accompanying us in our clinical on calls and rounds.

## Background

**Fig. 1:**
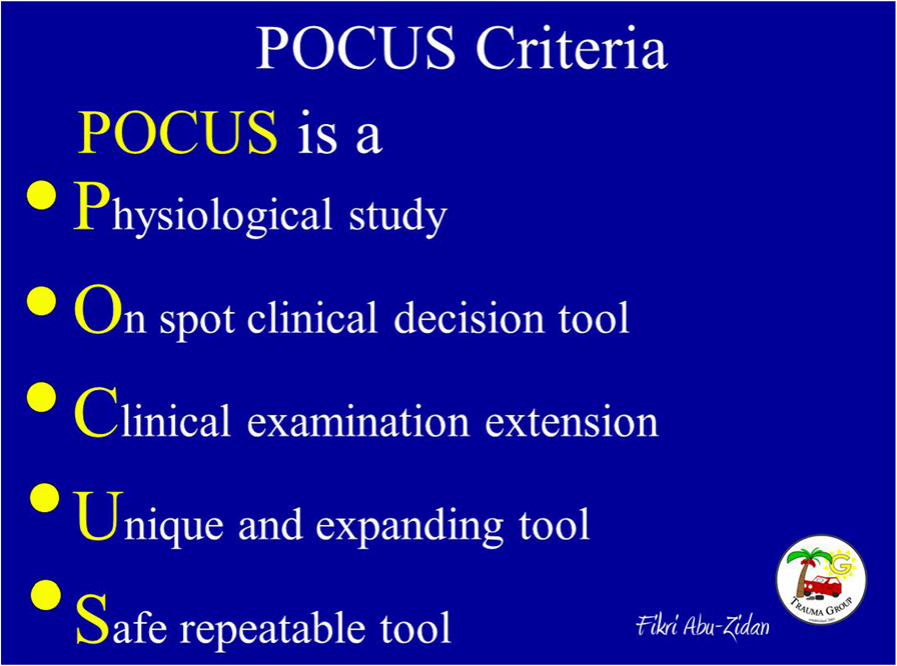
Major characteristics of point-of-care ultrasound (POCUS) which is performed by acute care physicians and makes it different from routine ultrasound examinations

Point-of-care ultrasound (POCUS) is a non-invasive diagnostic bedside tool which is fast, repeatable, accurate, and radiation-free. It is useful in critical decision-making [1, 2]. The use of POCUS by non-radiologists has developed over time. Initially, it was used to detect free intraperitoneal fluid in multiple trauma patients [3] and was termed as focused assessment sonography of trauma (FAST). As the skills and value of POCUS increased in the hands of non-radiologists, it developed into a life-saving diagnostic tool that is done by the treating physicians through the stages of diagnosis, resuscitation, operation, and postoperative critical care when managing sick patients [4]. Today, there is enough evidence to show that POCUS is a valuable tool for diagnosing gastrointestinal pathology even in the hands of non-radiologists [5]. Over the last 30 years, we have realized that POCUS is completely different from the routine radiological studies (Fig. 1). These differences include (1) POCUS is a *Physiological* study in which the shock status can be evaluated [6, 7], (2) it is an *On spot* clinical decision tool that helps in critical decision making in emergency situations within a very short time [8], (3) it is an extension of the *Clinical* examination and considered as the seventh sense of intra-abdominal inspection [9], (4) it is *Unique* with expanding indications to study different organs in a systematic approach at the same time [6], and finally, (5) it is *Safe* and repeatable and can be used exactly as a stethoscope in the hands of trained acute care physicians [6].

These significant advantages make POCUS valuable in many clinical settings including emergency departments, intensive care units, and operation theatres. With increased interest, training, and experience, POCUS will have a more pronounced role in diagnosing gastrointestinal pathology. This review aims to lay the basic principles of using POCUS in diagnosing intestinal pathologies so as to encourage residents and young colleagues to learn and master this important tool. It will cover the basic physics, technical aspects, and simple applications including detection of free fluid, free intraperitoneal air, and bowel obstruction. A more advanced and detailed review on specific intestinal pathologies will include appendicitis, epiploic appendagitis, acute diverticulitis, pseudomembranous colitis, intestinal tuberculosis, Crohn’s disease, and colonic tumours.

## Basic physics

**Fig. 2:**
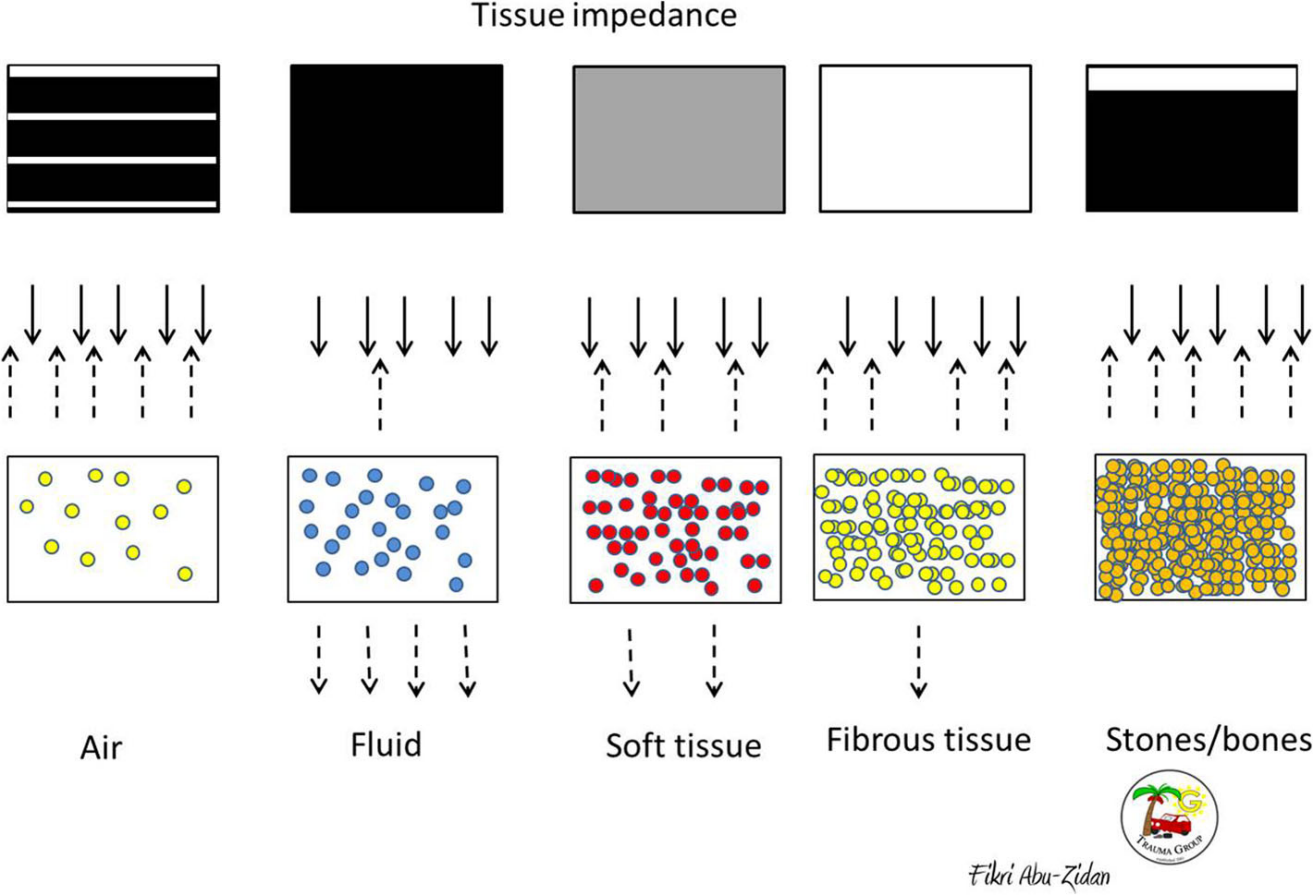
Denser materials reflect more ultrasound waves. As a result, fluid (like ascites) is black (anechoic), soft tissue (like the liver) is grey, fibrous tissue (like the diaphragm) is white without a shadow, and stones are white (hyperechoic) with a shadow. The air is very hyperechoic having reverberation artefacts

Understanding the basic physics of ultrasound and its artefacts is essential to interpret the ultrasound images, to avoid its pitfalls, and to reach an accurate bedside diagnosis. POCUS machines send high-frequency ultrasound waves (2–15 MHz) through their piezoelectric crystals, which are located in the probes, and then receive the reflected waves [10, 11] (Fig. 2). The two-dimensional (2D) brightness mode (B-mode) is the basic mode when performing POCUS [11]. This mode produces very thin black and white two-dimensional images (1 mm thick). These images can be modified by changing the frequency of the waves, the shape of the probe and its size, and the time in which the waves are emitted [12, 13].

Different media have different densities giving them different tissue impedance (Fig. 2). As the density increases, the particles of the media get more attached to each other, with an ability to reflect more ultrasound waves [14]. Accordingly, the particles will be more attached in stones/bones followed by fibrous tissue, soft tissue, fluid and finally gas consecutively (Fig. 2). As the tissue reflects more waves, it becomes more echogenic. Therefore, in the B-mode image, the fluid becomes black (anechoic), soft tissue becomes grey, fibrous tissue becomes white without a shadow (hyperechoic), and bones/stones become white with a shadow [15].

**Fig. 3:**
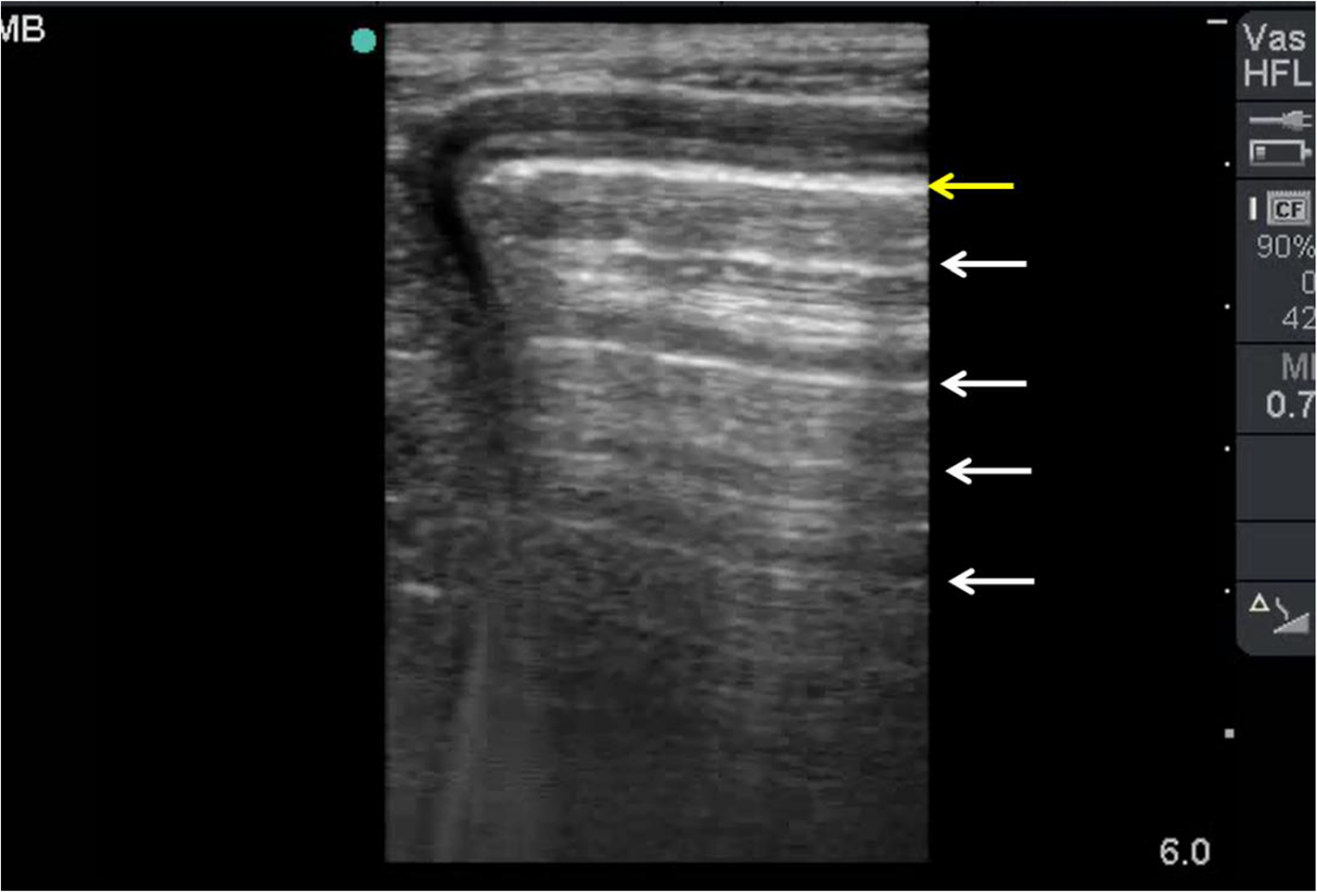
POCUS using a high-frequency linear probe for a gas-filled colon showing a reverberation artefact. This occurs when ultrasound waves bounce between the transducer and the gas. The gas is a very hyperechoic white line (yellow arrow). The reverberation lines (white arrows) represent the repetition of the reflective waves. The density of these lines becomes less as they go deeper while the distances between them are equal

Because of the fast movement of the gas particles, air is a very strong reflector for ultrasound waves. It gives a very hyperechoic shiny white lines that prevent visualization of deep structures. The deeper reverberation lines represent the repetition of the reflective waves. This occurs because ultrasound waves bounce between the transducer and the gas. The distance between these lines are usually equal [16] (Fig. 3). Nevertheless, the reverberation artefact is very useful to diagnose free intraperitoneal air (FIA). A very echogenic shiny line located directly under the abdominal fascia which does not move with respiration is diagnostic of FIA.

## Examination techniques

Low-frequency probes (2–5 MHz) have the stronger ability for tissue penetration while high-frequency probes (10–12 MHz) have less penetration ability but better resolution. As a result, studying deeper structures of the abdomen in obese patients (like detecting free intraperitoneal fluid) needs low-frequency probes while diagnosing superficial structures with high resolution (like acute appendicitis) needs high-frequency probes [11]. The low-frequency probes are used for wider and deeper views while the higher frequency probes are used for superficial structures with more details [12, 14].

**Fig. 4:**
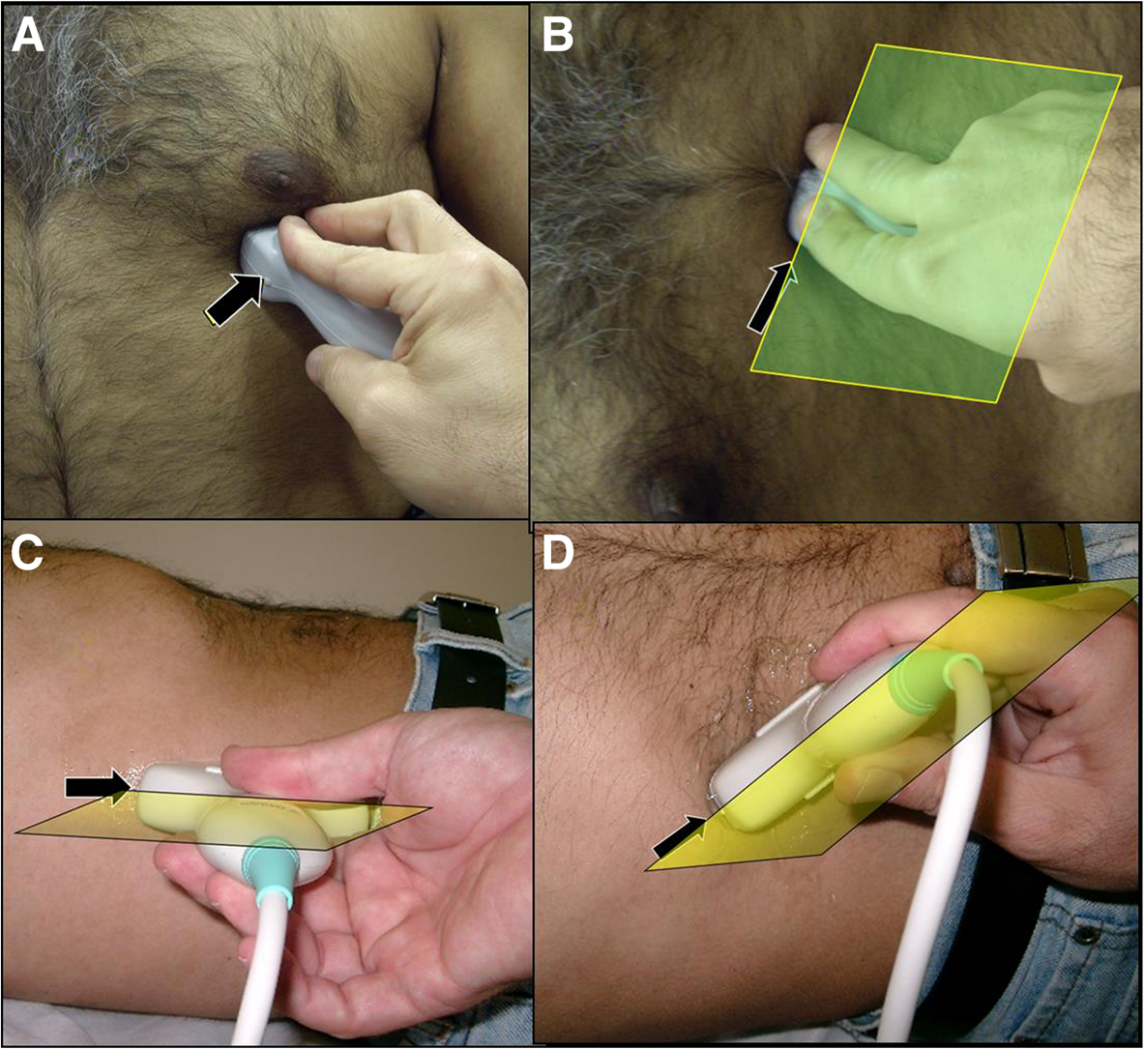
Acute care physicians use a global clinical ultrasound examination approach and may examine different anatomical regions in the same patient. The pointer of the ultrasound probe should be routinely directed proximally or to the right side of the patient even in echocardiography or oblique views. The figure demonstrates four different views, the cardiac apical four chamber view (**a**), the cardiac subcostal view (**b**), the coronal abdominal and thoracic view (**c**), and the oblique abdominal view (**d**). The marker is pointing proximally or to the right side of the patient

Acute care physicians use a global clinical ultrasound examination approach and may examine different anatomical regions, including the chest, abdomen, and extremities, at the same time if needed. Consequently, special considerations should be taken to observe the direction of the pointer of the ultrasound probe. It should be always directed towards the head or to the right side of the patient [12] (Fig. 4). The POCUS examination is a focused examination trying to answer a specific clinical research question which should be correlated with the clinical picture so as to maximize its value. Hence, the technique has to be individualized depending on the suspected pathology.

The operator should have a clear mental three-dimensional anatomical map with a clear plan before performing any radiological study. This would depend on the specific clinical question and the suspected gastrointestinal pathology. For example, if the colon is going to be examined, it has to be first located and then scanned. We always try to identify the ascending colon at the right flank (as recognized by its position and haustrations) and then follow the colon all the way down to the rectum [17]. If that was difficult, then the ascending colon can be located at the right upper quadrant, because it is more consistent, and then followed both proximally and distally [18].

The three “unhappy triad of intestinal ultrasound” include “too much pain, too much gas, and too much fat” [19]. These have to be reduced. For example, if retro-caecal appendicitis is suspected, analgesia may be needed, gentle compression on the bowel may move the gas of the caecum away, and if it was difficult to see the appendix from the anterior direction, then a trial locating the appendix from the right lateral side may be successful to detect a retro-caecal appendicitis. Ultrasound is operator-dependent, and these skills can be learned only by experience.

## Free fluid

POCUS can easily detect free intraperitoneal fluid with high accuracy [6, 20]. It can detect up to 10 ml of free fluid in trained hands [21]. Curvilinear low-frequency transducers (3.5–5 MHz) are usually used to detect free intraperitoneal fluid. The hepato-renal, peri-splenic, and pelvic regions are the three main dependent locations to detect free intraperitoneal fluid. The amount of detected fluid at a specific point of time will differ according to the anatomical location, rate of accumulation, and the patient’s position. In the supine position, peri-splenic space and Morrison’s pouch are the most common locations to detect free intra-abdominal fluid [21]. Trendelenburg and reverse Trendelenburg positions shift the fluid up to the hypochondrium or down to the pelvis, respectively. In addition, ultrasound can guide paracentesis of intra-abdominal fluid for further investigation with low risk of complications [22]. One of the limitations of POCUS is that it cannot differentiate between different types of fluid (urine, bile, ascites, or blood). It is only by clinical correlation or paracentesis that the type of fluid can be recognized. Furthermore, clotted blood is echogenic and can be missed easily if attention was not taken.

## Free intraperitoneal air

**Fig. 5:**
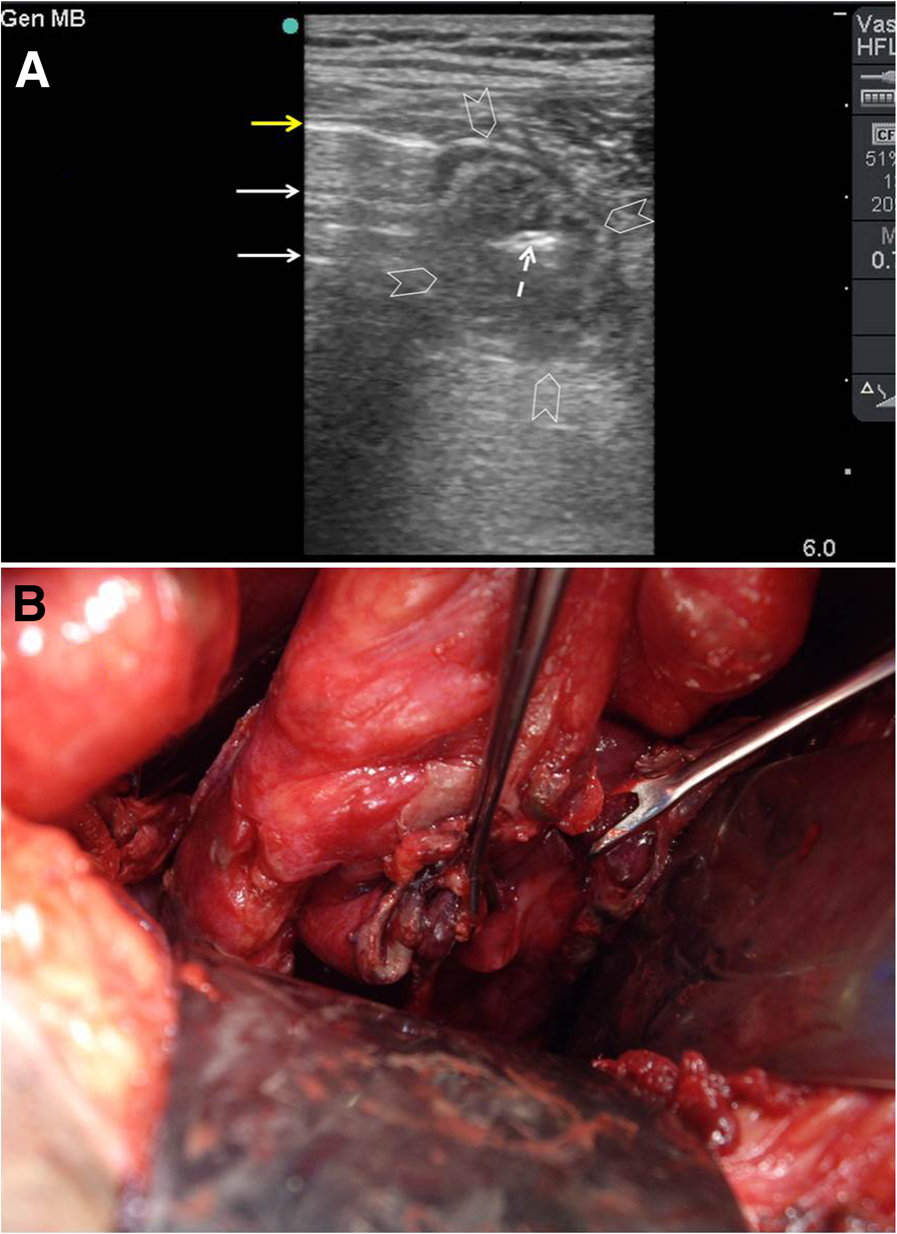
A 45-year-old man presented with suprapubic pain and dysuria. Abdominal examination showed guarding and tenderness in the suprapubic region. POCUS using a high-frequency linear probe (**a**) showed thickened inflamed small bowel (arrowheads) having gas within it (white dashed arrow). The significant free intra-peritoneal air was seen as an “enhanced peritoneal stripe sign” which is the hyperechoic white line located just under the abdominal fascia (yellow arrow). The reverberation lines (white arrows) represent the repetition of the reflective waves. Rectal examination detected a large self-inflicted rectal tear. Laparotomy confirmed the findings (**b**)

POCUS can accurately detect free intraperitoneal air (FIA) [23, 24]. There are three sonographic findings for FIA: enhanced peritoneal stripe sign, shifting phenomenon, and reverberation artefact [25]. In a supine patient, FIA moves anteriorly and can easily be found in the right upper quadrant (between the liver and the abdominal wall) and at the sub-hepatic region. Air is a strong reflector for the ultrasound waves giving increased echogenicity under the abdominal fascia (Fig. 5). This finding is called *enhanced peritoneal stripe sign* which can be changed by patient’s position (*shifting phenomenon*). The reverberation artefacts will hide the underlying organs. Differentiation between FIA and intraluminal gas is important. The hyperechogenic intraluminal gas of the bowel moves with respiration. However, FIA is directly located under the abdominal fascia and will not be affected by respiration [26].

Both linear and convex transducers can be used to detect FIA [27]. However, linear transducers (10–12 MHz) are more accurate in identifying the air near the abdominal surface because of their high resolution [26, 28]. The ultrasound exam should be first done when the patient is in the supine position during which the thorax should be elevated by 10–20 degrees [29]. In this position, the operator should focus on examining the midline and the right upper quadrant [26]. The transducer is placed longitudinally to the right paramedian epigastric location over the liver [29]. Some patients may need to be re-examined in the left lateral decubitus position. This may not be possible in many patients.

*Scissor manoeuvre* [30] is applied by using a linear transducer so as to demonstrate the air movement under compression. In this technique, FIA is identified between the liver and the abdominal wall. The operator applies compression in this area and suddenly releases it to show the air movement. In the compression phase, air will move away from the probe, and the FIA becomes less obvious. When compression is released, FIA will become clearer. Since more leaking air may accumulate over time, repeating the ultrasound examination is recommended so as to increase its accuracy [28].

## Intestinal obstruction

Intestinal obstruction is a common gastrointestinal emergency that needs rapid and efficient management. History, physical examination, and plain radiography are essential for its diagnosis [31]. The role of ultrasonography in diagnosing intestinal obstruction is recognized since nearly four decades [32]. Additionally, ultrasonography may help in detecting the cause and level of the obstruction [33].

The ultrasound examination should follow the anatomical path of the colon and small intestine. The normal bowel is seen as a circular hypoechoic structure (muscle layer) surrounding a mixture of hypoechoic (fluid), isoechoic (food), and hyperechoic contents (gas). The hypoechoic wall gets thinner during the relaxation period of peristalsis. The normal diameter of the bowel loops is up to 25 mm in the jejunum, up to 15 mm in the ileum, and up to 50 mm in the colon [31, 34]. POCUS provides answers to important clinical questions like: (1) Is there an obstruction? (2) Is the obstruction mechanical or functional? (3) Where is the location of the obstruction? (4) Is there ischaemia or necrosis of the bowel? and (5) What is the clinical progress of the patient who was treated conservatively? [35].

**Fig. 6:**
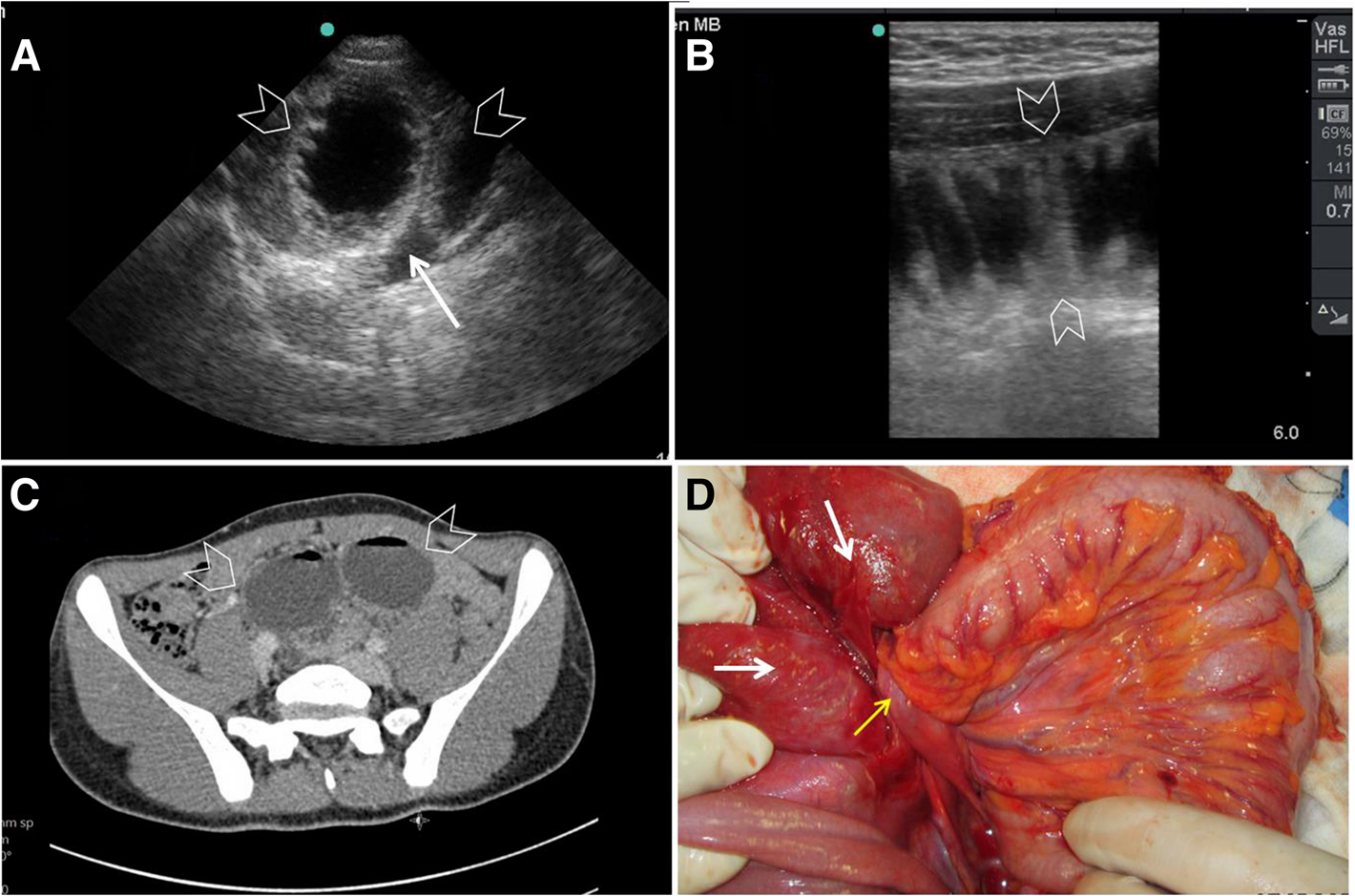
A 12-year-old boy presented with abdominal pain and vomiting. On examination, the lower abdomen was not distended, soft, but tender. Bowel sounds were reduced. POCUS examination revealed two non-functioning jejunal loops (arrowheads) (**a**–**b**) as evidenced by the valvulae conniventes of the loops. Abdominal CT scan confirmed the same findings (**c**). The patient did not respond to conservative management and needed surgery. Laparotomy (**d**) has shown the two jejunal loops (white arrows) obstructed by a congenital fibrous band (yellow arrow)

**Fig. 7:**
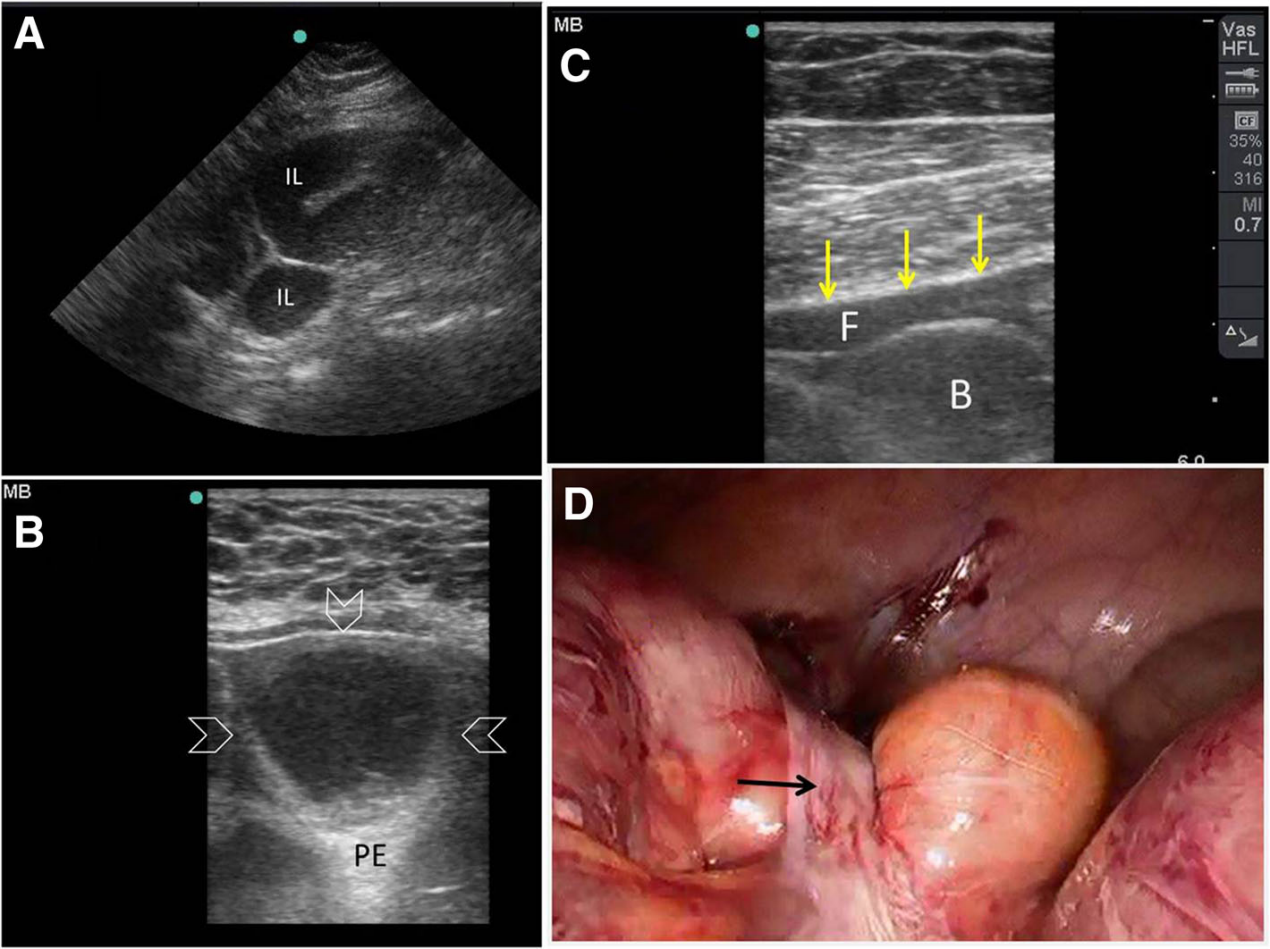
A 24-year-old woman presented with 1 day duration of abdominal pain, distension, vomiting, and constipation. She had a caesarian section 7 months ago. The abdomen was distended, soft, and non-tender. POCUS (**a**) using small print convex array probe (3–5 MHZ) showed active dilated ileal loops (IL) which was confirmed by the high-frequency linear probe (10–12 Mhz) (arrowheads) (**b**), PE = posterior enhancement artefact. Follow-up POCUS 12 h later using the linear probe (**c**) showed an increased amount of intraperitoneal fluid (F) between the bowel loops (B) and the abdominal fascia (yellow arrows). The patient underwent laparoscopic surgery (**d**) to release the adhesions (black arrow)

Ultrasound findings of small bowel obstruction include (1) increased loop dimensions, (2) increased wall thickness (more than 3 mm), (3) increased intestinal contents, (4) increased (to and fro) or decreased peristaltic movements, (5) enlarged and visible valvulae conniventes (more than 2 mm), and (6) collapsed colonic lumen [31, 35] . These findings are important to differentiate mechanical obstruction from functional obstruction. Dilated small bowel loops, full of gas and fluid, without peristalsis which is associated with colonic gas, fluid, or faeces should be considered as paralytic ileus. Diagnosing the location of the obstruction in the small bowel depends on the visibility of valvulae conniventes. These structures become prominent in the jejunal obstruction (Fig. 6a–d) and absent or rare in an ileal obstruction (Fig. 7a–d).

**Fig. 8:**
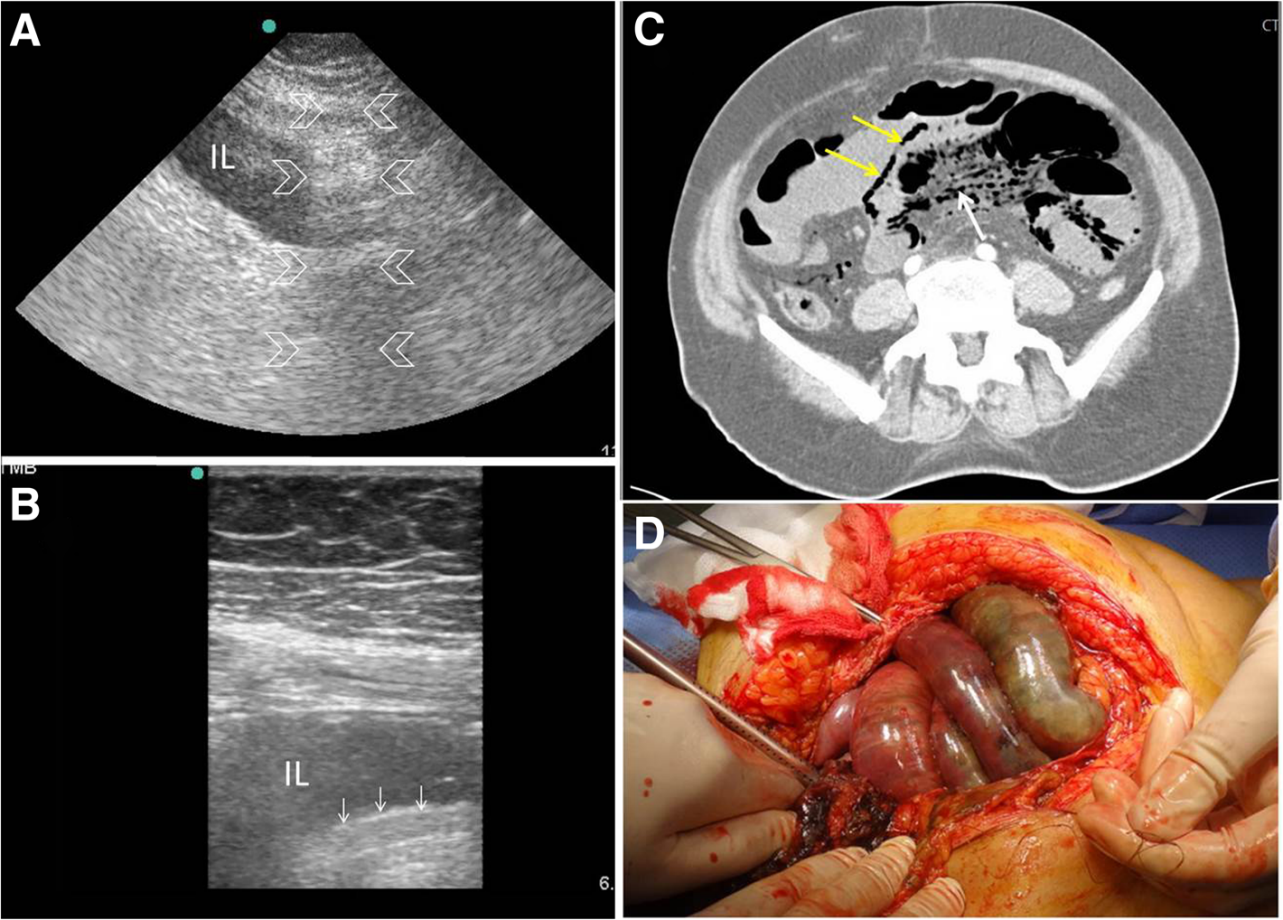
A 60-year old man who had atrial fibrillation developed abdominal pain of 24 h duration. He had a distended tender abdomen. Bowel sounds were negative. POCUS (**a**) was done using small print convex array probe (3–5 MHz). It was unexpectedly difficult because of obesity and unusual abdominal reverberation artefacts (arrowheads). The ileum (IL) was dilated and non-active. The high-frequency linear probe (10–12 Mhz) (**b**) confirmed these findings and detected gas within the bowel wall shown as tiny white dots (white arrows). CT angiography scan (**c**) showed superior mesenteric artery occlusion with massive bowel ischaemia. The small bowel loops had non-enhancing walls and pneumatosis intestinalis (yellow arrows). There was massive gas in the mesenteric vessels (white arrow). Laparotomy confirmed these findings (**d**). Clinical image, Courtesy of Dr Hussam Mousa, Department of Surgery, College of Medicine and Health Sciences, UAE University, Al-Ain, United Arab Emirates

It is also important to look for the cause of obstruction at the transit point between the dilated proximal loops and collapsed distal ones. Ultrasound is capable of diagnosing several pathologies including hernias, intussusception, ascariasis, foreign bodies, and tumours [33]. Bowel ischaemia reduces the bowel movements even when a mechanical obstruction exists (Fig. 8a–d). The bowel loop is considered as akinetic if the peristaltic movement is absent for more than 5 min [34]. The decision for early surgery, although clinical, is helped by the ultrasound findings of increased bowel wall thickness, decreased or absence of peristaltic movements, and presence of intraperitoneal fluid. Colour Doppler application may also provide information about the bowel wall perfusion [36].

## Acute appendicitis

Acute appendicitis is a common emergency pathology and may perforate in one-third of the cases if the diagnosis was delayed [37]. Multiple imaging modalities are useful in the diagnosis of acute appendicitis. However, ultrasound is recommended as the first modality of choice for all age groups, especially in children because of its safety [37–39]. Acute care physicians showed high accuracy in diagnosing acute appendicitis [40].

**Fig. 9:**
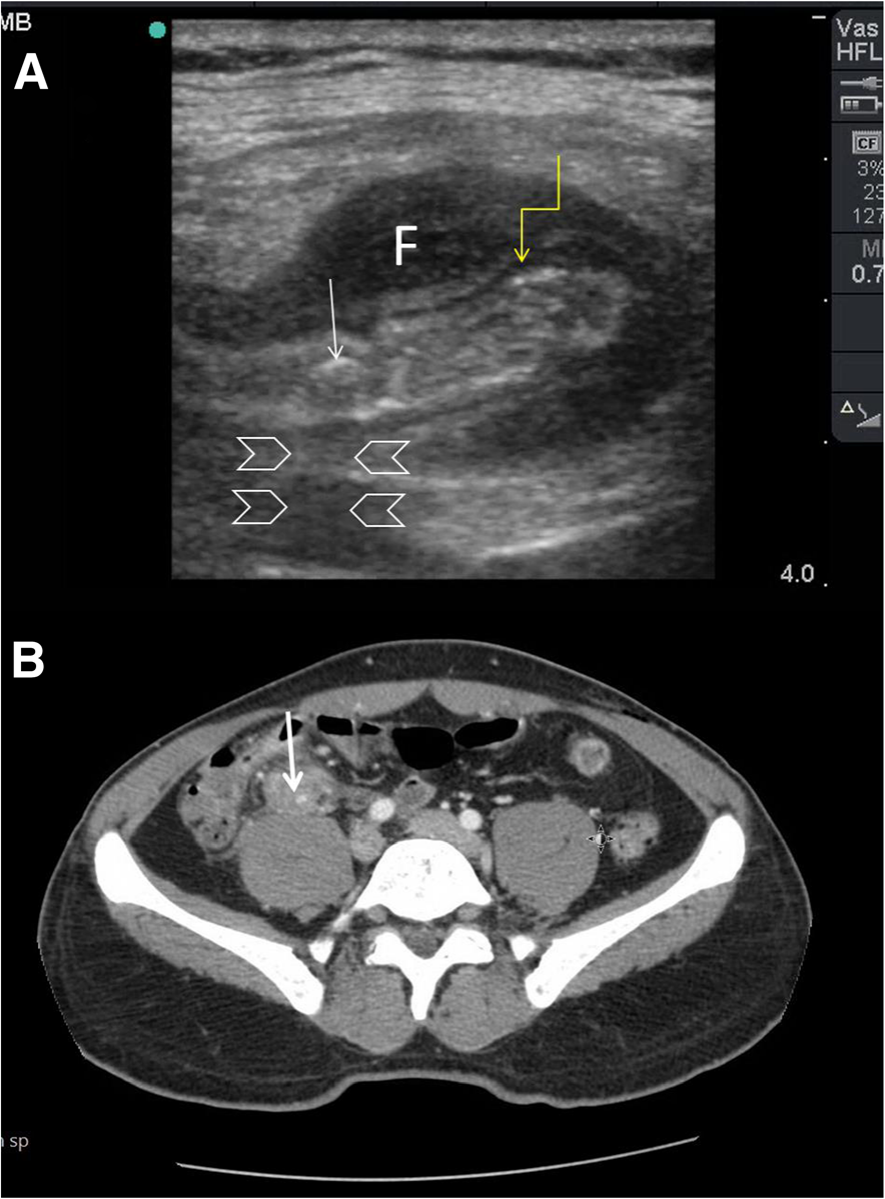
A 40-year-old man presented complaining of fever and pain in the right iliac fossa of 5 days duration. A hard tender mass was felt in the right iliac fossa. CRP was high. POCUS study (**a**) using a portable ultrasound machine and a high-frequency linear probe has shown a thickened appendicitis with oedema in the muscularis propria (yellow arrow) surrounded by fluid (F). There was a faecolith (white arrow) with a shadow behind it (arrowheads). Abdominal CT scan with intravenous contrast has confirmed the same findings (**b**). Interval appendectomy which was performed two months later confirmed the diagnosis

The ultrasound examination is applied in the supine position using a high-frequency linear probe. The graded compression technique is the standard technique [38, 41]. This technique aims to reach deeper penetration by compressing and pushing the air away so as to visualize the appendix. The normal small bowel is compressible with air inside it while acute appendicitis is non-compressible and rarely has air inside it. The normal mean diameter of the appendix ranges between 4.4 and 5.1 mm [42]. Consequently, a diameter larger than 6 mm is suggestive of acute appendicitis in the proper clinical setting. Trout et al. [43] showed that an appendix diameter of 6 to 8 mm and more than 8 mm had the highest accuracy in diagnosing appendicitis (65%, 96% respectively), while there was only 2.5% of appendicitis having a diameter of less than 6 mm. There are other direct findings for diagnosing acute appendicitis including the target sign, appendicolith, and hypervascularity with Doppler ultrasound (Fig. 9). Other indirect findings include free fluids around the appendix, abscess formation, increased mesenteric fat echogenicity, enlarged local mesenteric lymph nodes, and increased peritoneal thickness [38]. Ultrasound results in acute appendicitis can be affected by the body mass of the patient, the thickness of the body wall, pain score as well as the experience of the operator.

## Epiploic appendagitis

Primary acute epiploic appendagitis is relatively uncommon. Its diagnosis and management continues to be a challenge. Depending on its anatomical site, it may mimic acute appendicitis, acute diverticulitis, or acute cholecystitis [44]. Before the era of modern diagnostic modalities, its preoperative diagnosis was difficult and it was reached only after surgery. New diagnostic modalities, like abdominal ultrasound and computed tomography (CT) scan, made it possible to reach an accurate preoperative diagnosis giving an option for conservative management [45].

**Fig. 10:**
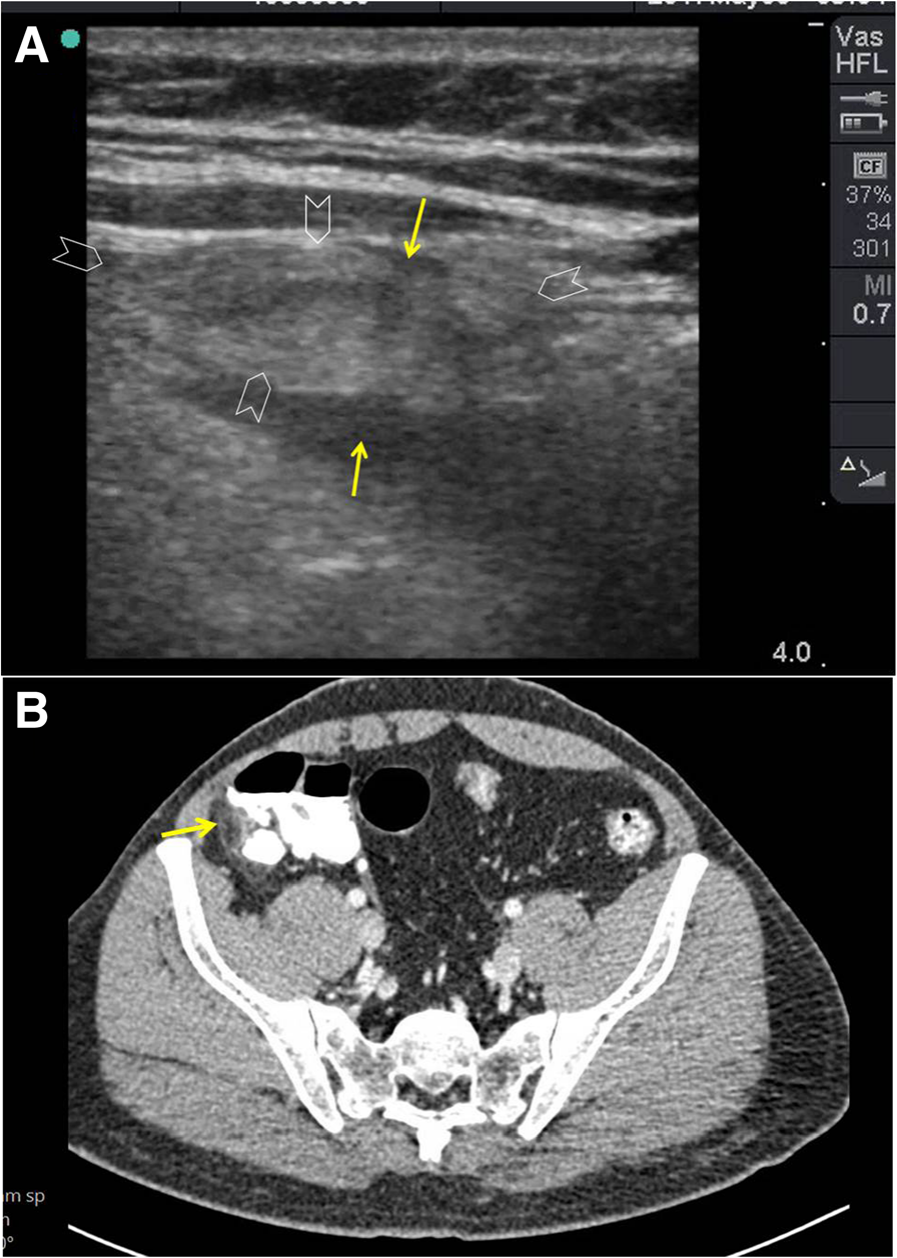
A 36-year-old man presented with right iliac fossa pain. The patient was afebrile. There was no leukocytosis and C-reactive protein was normal. The abdomen was soft but tender. POCUS (**a**) using a high-frequency linear probe showed a hyperechoic non-compressible ovoid mass adherent to the colonic wall at the maximum tenderness point (arrowheads) which was surrounded by fluid (yellow arrows). Abdominal CT scan with intravenous contrast (**b**) confirmed the diagnosis of epiploic appendagitis (yellow arrow)

The normal appendices epiploicae are not seen by abdominal ultrasound or CT unless surrounded by intraperitoneal fluid [46]. Ultrasound of an acute epiploic appendagitis shows as a small hyperechoic non-compressible ovoid mass adherent to the colonic wall, which is frequently surrounded by hypoechoic hallo at the maximum tenderness point [47, 48] (Fig. 10). Some consider ultrasound to be the key imaging modality for the diagnosis of pathological twisted appendix epiploicae [49].

## Diverticulitis

In 2006, Liljegren et al. performed a systematic review comparing ultrasound, CT scan, and magnetic resonance imaging for diagnosing acute colonic diverticulitis. They concluded that ultrasound should be the method of choice for diagnosing acute diverticulitis supported by evidence at that time [50]. In 2008, Lameris et al. [51] did another systematic review that compared graded compression ultrasound with computed tomography. They included six ultrasound and eight CT scan studies having more than 600 patients in each modality. They found that the sensitivity and specificity of ultrasound and CT scan are statistically similar in diagnosing acute colonic diverticulitis (92% and 90% for ultrasound compared with 94% and 99% for CT scan consecutively). This was supported by a large prospective study of 802 patients presenting with acute abdomen [52]. Ultrasound and CT scan had high sensitivity and specificity in diagnosing colonic diverticulitis. Yet, they did not change the management of patients. Ultrasound alone had a sensitivity of 91% and a specificity of 100% while CT scan had a sensitivity of 95% and a specificity of 99% in diagnosing colonic diverticulitis. Adding CT scan and ultrasound together increased the diagnostic sensitivity and specificity to 100%.

A recent meta-analysis performed by Andberg et al. [53] has shown that ultrasound and CT have comparable results in diagnosing diverticulitis. However, CT scan has the advantage of higher specificity and the ability to identify alternative diagnoses. Finally, the recent guidelines of the World Society of Emergency Surgery, which was published in 2016, accepts ultrasound as an alternative in the initial evaluation of patients with suspected acute left colonic diverticulitis. Moreover, a step-up approach starting with ultrasound and performing CT after an inconclusive or negative ultrasound study was recommended [54]. This approach would reduce the exposure to radiation.

**Fig. 11:**
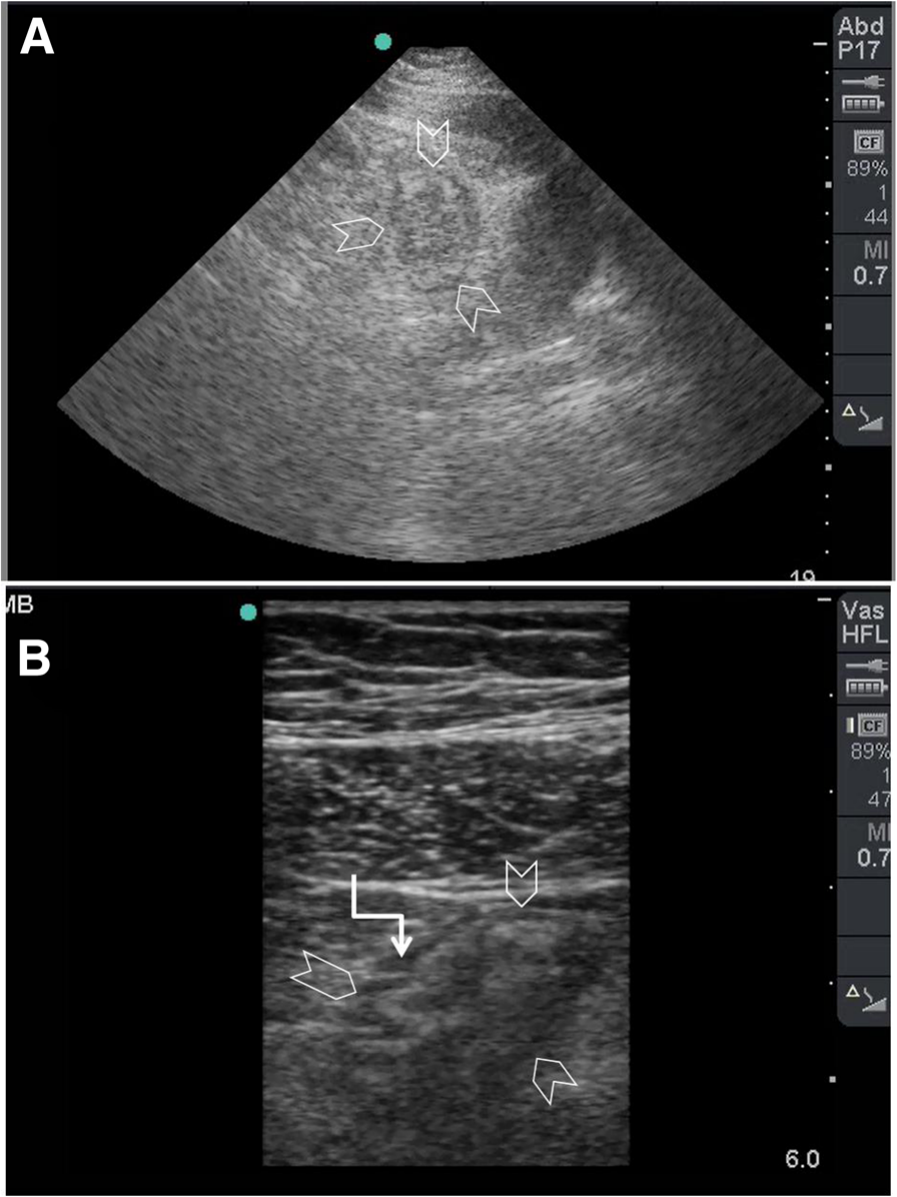
A 32-year-old man presented with left lower quadrant (LLQ) abdominal pain of 5 days duration. Abdominal examination revealed a tender mass in the LLQ. The patient had leukocytosis and raised CRP. POCUS study (**a**) using a portable ultrasound machine and a small print convex array probe with a frequency of 3–5 MHz has shown a rounded mass (arrowheads). A more detailed image (**b**) using a high-frequency linear probe showed that the mass (arrowheads) had a rounded folded edge giving it the classical *saw-tooth pattern.* The hypoechoic area represents the muscularis propria (arrow)

Generally, abnormal ultrasound findings of the inflamed bowel include (1) thickened wall of more than 4 mm, (2) non-compressibility and (3) loss of peristalsis [17, 18]. Using the graded compression technique, the diagnosis of acute colonic diverticulitis can be made by finding a hypoechogenic mural thickening of the colonic wall (more than 4 mm) and a non-compressible target sign in the transverse section. The hypoechoic area represents the muscularis propria [17, 19] and is caused by inflammation, oedema, and hypertrophy [18]. The affected inflamed colonic segment may have a rounded folded edge giving it the classical *saw-tooth pattern* (Fig. 11). The layers of the colonic wall are usually preserved in diverticulitis compared with malignant tumours. [17]. The surrounding inflamed fat of the mass will be echogenic [18]. Besides, POCUS may detect complications of diverticulitis depending on its stage which may include abscess formation, free intraperitoneal fluid, and free intraperitoneal air which can be correlated with the classification of acute diverticulitis [28, 55, 56].

## Pseudomembranous colitis

Ultrasound is recommended as a screening tool in patients suspected to have pseumembranous colitis especially in patients who cannot be moved to the CT scan. Although the ultrasound findings in pseudomembranous colitis are not pathognomonic, their presence combined with the clinical picture should raise the suspicion of the diagnosis [57–59]. It is advised to use a high-frequency linear probe of 10–12 MHZ with gentle abdominal compression to study the details of the colonic wall. The presence of intraperitoneal fluid, thickened wall of the colon, and minimum intraluminal gas facilitates the ultrasound examination in this condition [57].

Ultrasound findings of pseudomembranous colitis in severe cases include a thickened colonic wall with heterogeneous echogenity and narrowing of the colonic lumen [18]. The echogenic intraluminal contents of the large bowel become clearer because of the scarcity of intraluminal gas. Pseudomembranes can be seen as hyperechoic lines covering the mucosa [57–59]. These findings will give an “accordion sign appearance” similar to a CT scan. The sonographic colonic wall thickness of more than 1 cm and an accordion sign in the proper clinical setting is suggestive of *Clostridium difficile* colitis.

**Fig. 12:**
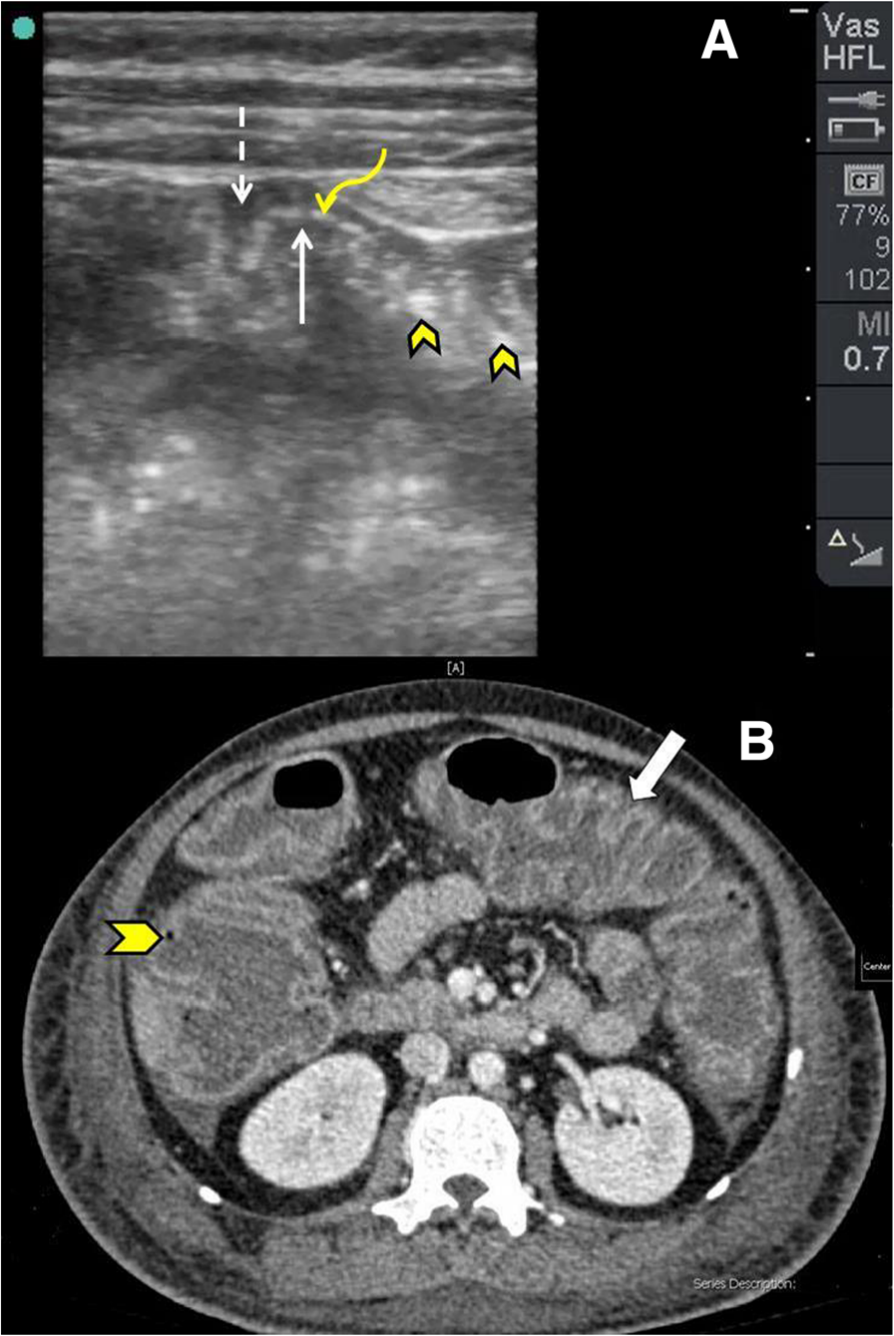
POCUS study (**a**) using a high-frequency linear probe in multi-trauma patients who developed diarrhoea 2 weeks after starting antibiotic administration. The colonic wall is thickened with three distinct layers, mucosa (white arrow), submucosa (curved yellow arrow), and muscularis propria (interrupted arrow). Hyperechoic lines are seen covering the mucosa (arrowheads). These can be either pseudomembranes or air bubbles. These findings give the classical accordion sign of pseudomembranous colitis. Abdominal CT scan with intravenous contrast (**b**) which was performed at the same time shows the same accordion sign (white arrow). CT scan has the advantage of showing the hyperaemic mucosa with air bubble in the colonic wall (arrowhead)

In early stages of pseudomembranous colitis, the texture of the wall of the colon is preserved. The hypoechoic oedematous mucosa and muscularis propria will be thickened. The echogenic submucosa is sandwiched between them. The submucosa may have numerous gaps indicating penetration of the infection into deeper structures (Fig. 12). Intraperitoneal free fluid is seen in more than 70% of the cases [57–59]. Additionally, ultrasound may detect free intraperitoneal air when bowel perforation is present [28].

## Intestinal tuberculosis and Crohn’s disease

The differential diagnosis between intestinal tuberculosis and Crohn’s disease can be extremely difficult. Intestinal tuberculosis involves mainly the terminal ilium and caecum while Crohn’s disease involves the small bowel and ascending colon [56]. Lim et al. [60] studied 45 cases of ileocaecal tuberculosis in whom ultrasound showed thickening of the terminal ilium and caecum in 43 patients (96%). Out of eight patients who had Crohn’s disease, seven (88%) had diffuse or mural thickening of the small bowel and/or colon [60].

**Fig. 13:**
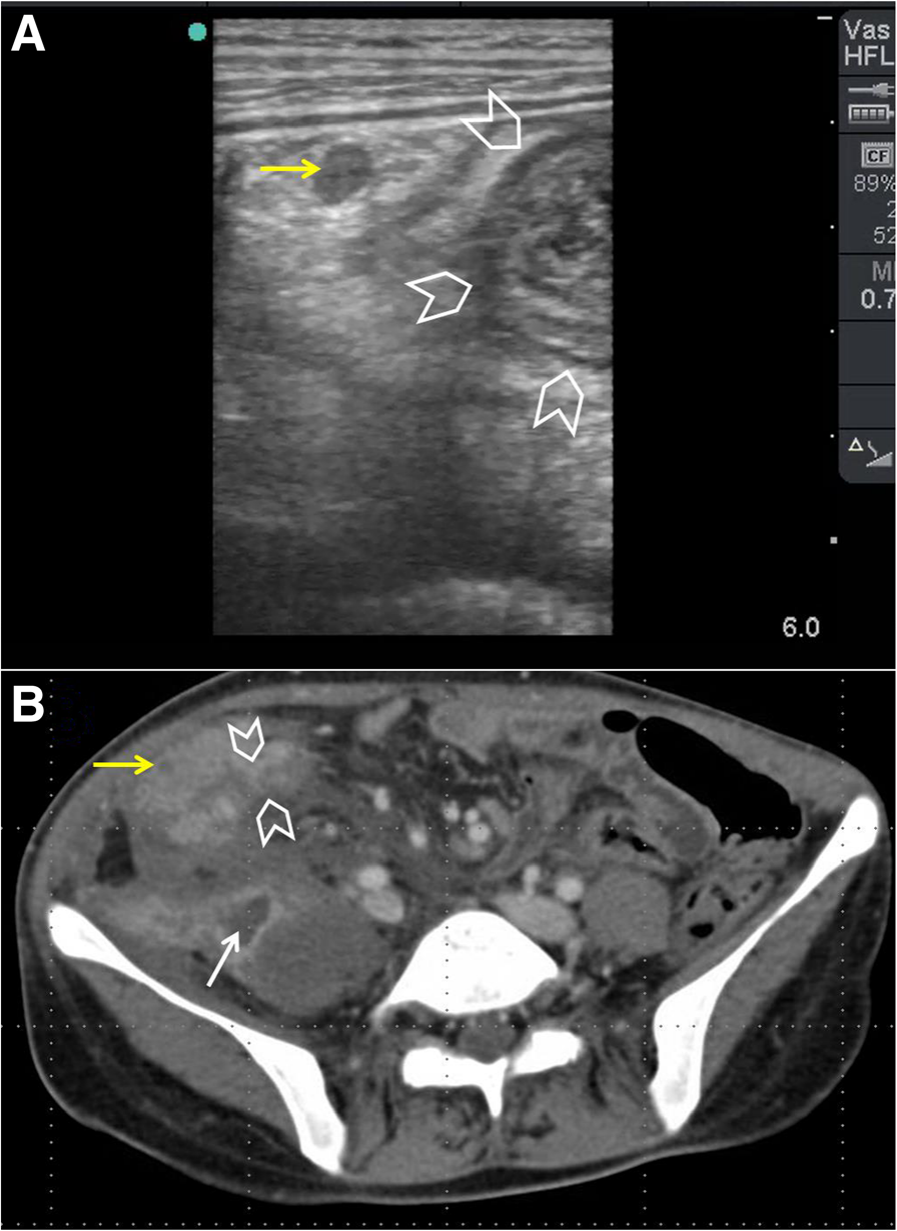
A 30-year-old woman presented with a tender right iliac fossa mass of 4 months duration. The mass was fixed and hard. POCUS (**a**) using a high-frequency linear probe showed matted thickened inflamed small bowel (arrowheads) and regional lymph nodes (yellow arrow). Abdominal CT scan with intravenous contrast (**b**) confirmed the presence of the thickened bowel (arrowheads) and the enlarged lymph nodes (yellow arrow). There was an ileo-psoas abscess (white arrow). Biopsies through a colonoscopy and an interferon test were non-conclusive. The patient was started on anti-tuberculous treatment as a therapeutic diagnosis

Intestinal tuberculosis may have three forms including (1) ulcerative type, (2) hypertrophic type, and (3) ulcero-hypertrophic type [61, 62]. Ileocaecal tuberculosis is usually of the hypertrophic type which makes it easy to evaluate by ultrasound. In early stages, ultrasound can show thickening of the wall of the terminal ilium and caecum (Fig. 13). With advanced disease, the medial wall of the caecum and ileocaecal valve gets more irregular thickening [61]. Ultrasound findings in abdominal tuberculosis may also include generalized or localized ascites with thin mobile septa, thick omentum and peritoneum, and lymphadenopathy [61–64].

**Fig. 14:**
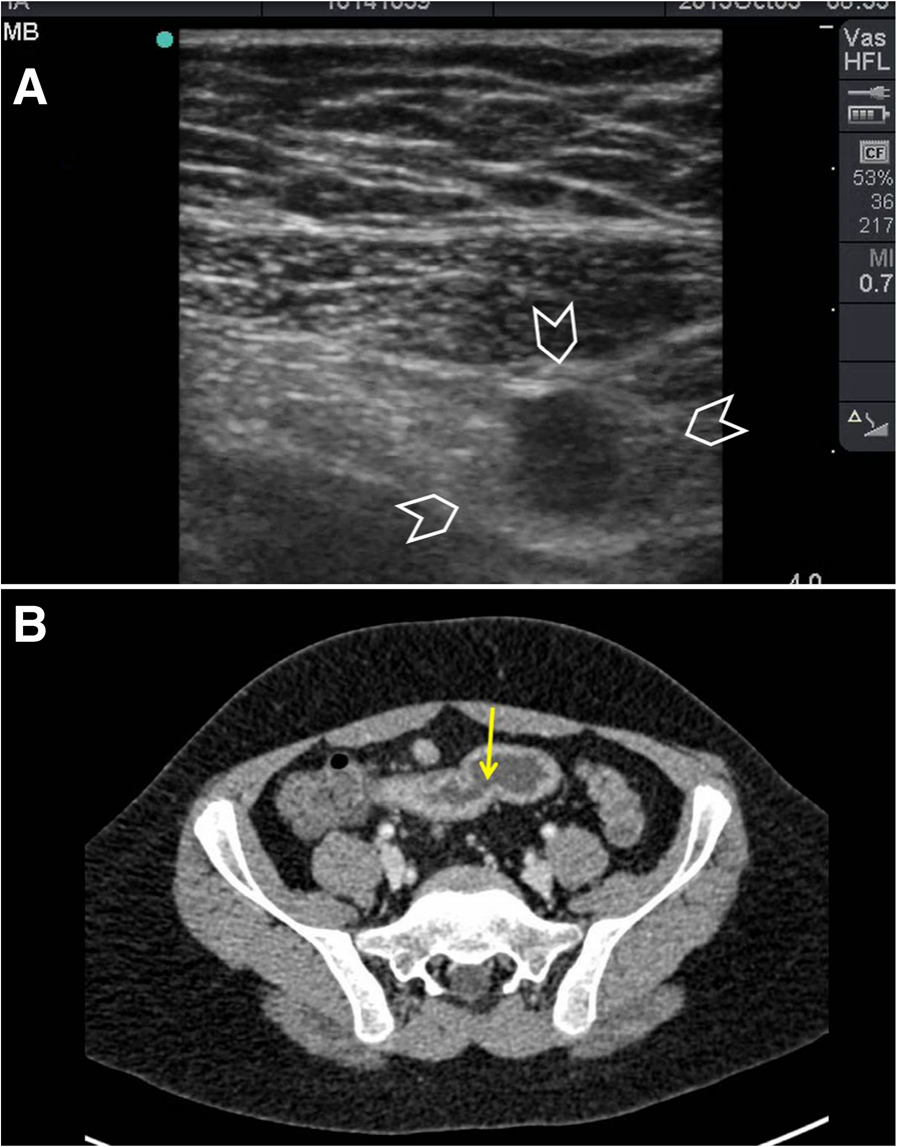
A 14-year-old boy presented with pain in the right iliac fossa of 1 month duration associated with diarrhoea. His abdomen was soft and tender at the right iliac fossa. POCUS (**a**) using a high-frequency linear probe of the right iliac fossa showed a thickened hyperechoic non-compressible small bowel (arrowheads). The bowel layers could not be seen. Crohn’s disease was suspected. CT with intravenous contrast (**b**) showed a thickened constricted terminal ileum suggestive of Crohn's disease (yellow arrow)

During the acute phase of Crohn’s disease, the thickened submucosa of the bowel will be hyperechoic because of lymphoedema (Fig. 14) while in the chronic phase the wall will be thickened and hypoechoic [18]. The adjacent fat will be echogenic because of the trans-mural inflammation of the bowel [18]. Adjacent lymph nodes are occasionally visualized [65]. Ultrasound may also demonstrate an adjacent fistula or abscess [18]. The lumen of an inflamed bowel is usually empty or near empty which makes the ultrasound study easier [60] (Fig. 14).

CT scan is superior to ultrasound in evaluating the extent and type of abdominal tuberculosis including lymphadenopathy [63, 64, 66]. A recent systematic review and meta-analysis compared the accuracy of CT scan findings in differentiating intestinal tuberculosis from Crohn’s disease [67]. This study compared 417 patients having Crohn’s disease with 195 patients having intestinal tuberculosis. Necrotic lymph nodes had a sensitivity of only 23% for diagnosing intestinal tuberculosis while both comb sign and skip lesions had a sensitivity of more than 80% for diagnosing Crohn’s disease. Still, it is important to stress that the radiological findings including wall thickening are non-specific in both intestinal tuberculosis and Crohn’s disease and a microbiological or histopathological confirmation should be obtained by endoscopy and biopsy [56, 62, 65, 68].

A definite diagnosis can be reached in abdominal tuberculosis in about 80% of the patients despite every effort made. Therapeutic diagnosis should then be tried in the suspected non-conclusive remaining 20% of the cases. The majority will have a rapid response to anti-TB treatment, usually within 2 weeks [64]. Starting patients who have active abdominal tuberculosis on steroids on the assumption that it is Crohn’s disease will worsen the clinical picture and even lead to death [64, 69]. The prevalence of the disease in a setting should be considered and caution should be adopted before starting steroids.

## Colonic tumours

**Fig. 15:**
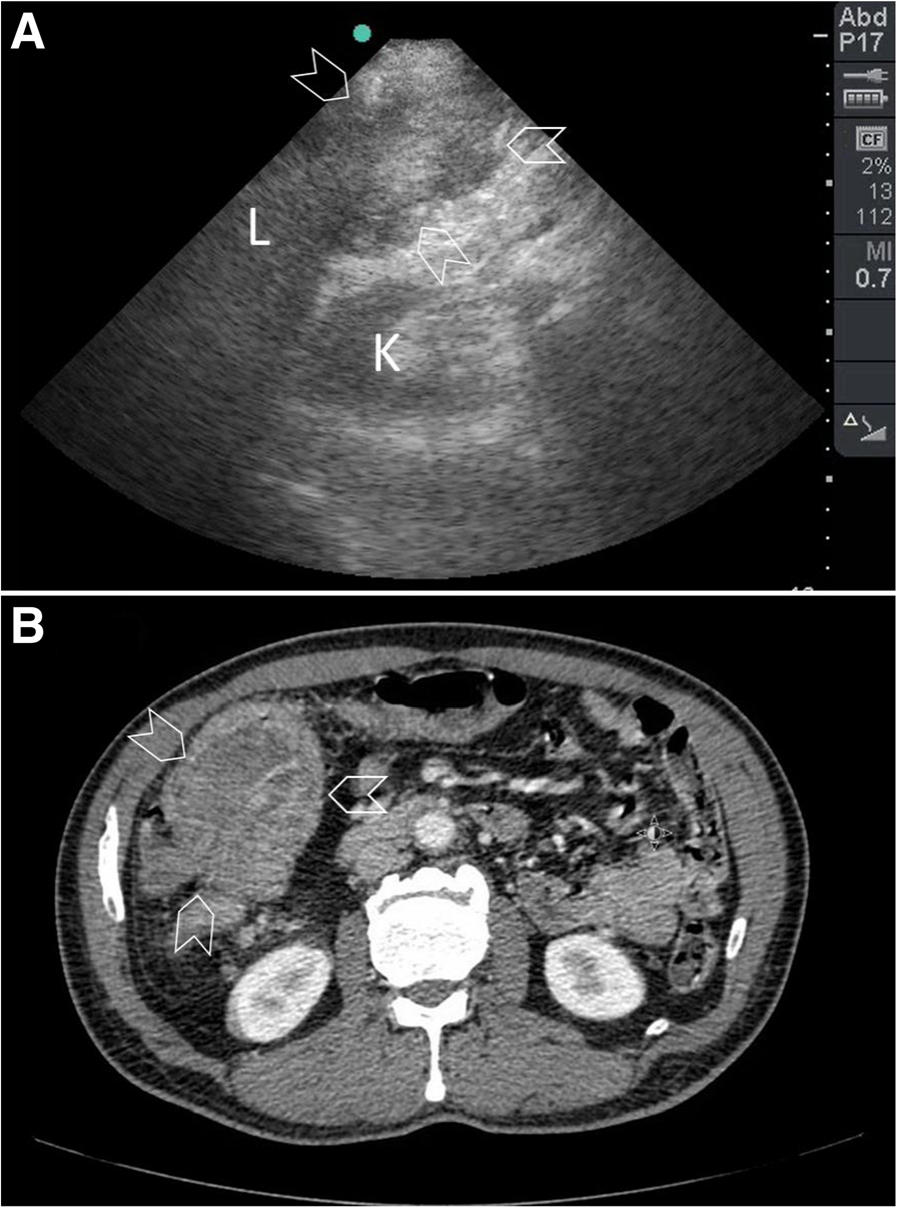
A 57-year-old man presented with abdominal pain and diarrhoea of 10 days duration. On abdominal examination, there was a mobile mass in the right upper quadrant. The abdomen was soft and not distended. The patient was anemic. POCUS study (**a**) using a portable ultrasound machine and a small print convex array probe having a frequency of 3–5 MHz has shown an irregular mass in the right colon without a lumen just under the liver and 10 cm long (arrowheads). A clinical diagnosis of right colonic malignancy was suspected. CT scan with intravenous contrast (**b**) has shown the same findings (arrowheads). The patient had a right hemicolectomy. The tumour was confirmed to be a poorly differentiated colonic adenocarcinoma. L liver, K kidney

Malignant colonic tumours can be either annular (constricting the lumen due to mural thickening) or polypoid (protruding into the lumen of the colon). They may fungate either inside or outside the colonic wall. They may be large (up to 10 cm) having an irregular or lobulated contour [56] (Fig. 15). Annular tumours are usually hypoechoic with central hyperechoic lines representing air [18].

The layers of the colonic wall are commonly destroyed in malignant tumours compared with diverticulitis [17]. In inflammatory diseases, the mural thickening is usually more uniform, thinner, and involves a longer segment of the bowel compared with malignancy [56]. Ripollés et al. [70] have recently studied the diagnostic accuracy of ultrasound to differentiate between diverticulitis and malignancy. They retrospectively compared 50 patients having diverticulitis with 41 patients having colon cancer. In their study, loss of the bowel wall stratification had a sensitivity of 92% and a specificity of 94% in diagnosing malignancy.

Ultrasound can miss early cancer or small tumours. Accordingly, it cannot be used as a screening tool. Nonetheless, it is useful to evaluate masses, hepatic lesions, the presence of ascites, or lymph nodes. Presence of lymph nodes has a very low sensitivity (around 30%) and a high specificity (98%) for identifying malignancy [70, 71].

## POCUS training

The results of POCUS depend on three factors: the operator, the ultrasound machine, and the patient. Acute care physicians should be trained and have their own proper ultrasound machines. POCUS can help clinicians improve their diagnostic skills, accuracy, confidence, and critical decision-making at the bedside [2]. It gives the clinician an advantage of direct application, interpretation, and provision of bedside decisions and care without delay. It can be repeated many times without the risk of radiation. Moreover, it is cheaper than computerized tomography or magnetic resonance imaging. Therefore, implementing POCUS training in medical programs including medical schools is spreading [72]. We are actually advocates of POCUS training for all our undergraduate medical students because we think it will become the stethoscope of the future [73].

POCUS is already used by various acute care physicians including surgeons, emergency physicians, and critical care physicians who have the same common language [74]. Because the results of ultrasound are operator-dependent, training for clinicians, who did not receive formal ultrasound training in their medical school or residency is essential. This will help them reach the desired clinical competency. This training may include diagnostic, procedural, screening applications, or combinations of all of these depending on the clinician’s own needs. Training and credentialing is a hallmark of a successful use of POCUS in patients who need critical decision-making [20, 75–77].

## Conclusions

Point-of-care ultrasound (POCUS) is an extension of the clinical examination when used by acute care clinicians in evaluating an acute abdomen. It is a unique safe diagnostic tool that can be used repeatedly on the bedside of sick patients. Deep understanding of basic physics of ultrasound and its artefacts is the first step in mastering POCUS. This helps both in reaching accurate POCUS diagnosis and avoiding its pitfalls. Detailed and accurate POCUS findings of specific intestinal pathologies can be obtained by trained acute care physicians. It is a great advantage to incorporate the POCUS findings directly with the clinical findings by the same operator so as to reach timely proper diagnosis and management.

## Abbreviations

2D: Two-dimensional
B-mode: Brightness mode
FAST: Focused assessment sonography of trauma
FIA: Free intraperitoneal air
POCUS: Point-of-care ultrasound
TB: Tuberculosis
